# Rural Hospital Bypass by Patients With Commercial Health Insurance

**DOI:** 10.1001/jamanetworkopen.2025.55017

**Published:** 2026-01-22

**Authors:** Jessica Y. Chang, Caitlin E. Carroll

**Affiliations:** 1 Division of Health Policy and Management, University of Minnesota School of Public Health, Minneapolis; 2 Health Care Cost Institute, Washington, DC

## Abstract

This cross-sectional study examines rural hospital bypass among commercially insured patients, including variation in bypass by rurality and clinical condition, and hospital payments generated by patients who bypassed their nearest hospitals.

## Introduction

Rural hospital bypass occurs when rural residents receive care at hospitals other than their nearest hospital. These patients may benefit from higher-quality treatment and improved health outcomes (eg, if bypass is driven by clinically appropriate referrals for specialty care). However, bypass reduces payments to rural hospitals and can erode profitability, making it difficult for hospitals to serve patients who need local services.^1^ For hospital finances, bypass by commercially insured patients is concerning because commercial insurers typically pay more than public insurers for the same services. Although half of rural residents are commercially insured, evidence on their bypass patterns is scarce.^2,3,4,5,6^ In this study, we examined rural hospital bypass among commercially insured patients, including variation by rurality and clinical condition, and hospital payments generated by patients who bypassed their nearest hospitals.

## Methods

In this cross-sectional study, we used commercial claims from the Health Care Cost Institute to study hospitalizations of nonelderly rural residents with employer-sponsored insurance between January 1, 2012, and December 31, 2021. Rural areas included nonmetropolitan counties and zip codes. We excluded newborn hospitalizations and interfacility transfers (eAppendix in Supplement 1).

Bypass was measured as the share of patients who (1) were hospitalized at any hospital other than their nearest hospital (by driving distance) and (2) bypassed their nearest hospital plus all rural hospitals within 30 miles, following previous work.^5^ For each measure, we calculated payments made to receiving hospitals for patients who bypassed the relevent set of 1 or more nearby hospitals. We also assessed bypass rates by rurality (noncore rural, small town, micropolitan, and metropolitan) and clinical category (diagnostic groups plus nonemergent admissions) (eAppendix in Supplement 1). The study was approved by the institutional review board of the University of Minnesota, which waived the need for informed consent owing to use of deidentified data. We followed the STROBE reporting guideline and used Stata, version 18, for statistical analysis.

## Results

This study included 2 166 054 hospitalizations of rural residents, including 1 433 500 (66.6%) in which the patient bypassed their closest hospital (Table). Bypass hospitalizations generated $34.9 billion in payments to receiving hospitals. Bypass rates grew from 2012 to 2019, then declined during the COVID-19 pandemic (Figure). Bypass rates varied by rurality (79.0% in noncore areas vs 57.9% in micropolitan areas) and clinical category. Bypass rates for the most common diagnostic groups—pregnancy-related and musculoskeletal—were 60.6% and 79.1%, accounting for $3.1 billion and $7.1 billion in payments, respectively. The bypass rate for nonemergent admissions was 77.4%.

**Table.** Rates of Rural Hospital Bypass by Commercially Insured Patients and Payments Generated by Bypass Hospitalizations, by Patient Rurality and Clinical Category

Variable	No. of hospitalizations	Bypass of closest hospital		Bypass of closest hospital and all rural hospitals within 30 miles	
		Hospitalizations, No. (%)^a^	Payment in billions, $^b^	Hospitalizations, No. (%)^a^	Payment in billions, $^b^
Total	2 166 054	1 443 500 (66.6)	34.9	1 231 015 (56.8)	31.4
Zip code rurality^c^					
Noncore	273 440	216 049 (79.0)	4.9	180 431 (66.0)	4.3
Small town	564 298	433 855 (76.9)	9.9	362 244 (64.2)	8.8
Micropolitan	1 219 009	706 348 (57.9)	18.1	602 039 (49.4)	16.3
Metropolitan^d^	109 307	87 248 (79.8)	2.0	86 301 (79.0)	2.0
Clinical category^e^					
Diagnostic group					
Nervous system	94 619	76 297 (80.6)	2.1	69 614 (73.6)	2.0
Respiratory system	155 961	80 316 (51.5)	1.7	65 447 (42.0)	1.4
Circulatory system	197 216	143 990 (73.0)	5.4	126 170 (64.0)	4.9
Digestive system	191 096	113 851 (59.6)	2.2	96 790 (50.6)	2.0
Hepatobiliary system and pancreas	71 974	41 151 (57.2)	0.8	34 783 (48.3)	0.7
Musculoskeletal system	250 303	197 880 (79.1)	7.1	175 273 (70.0)	6.4
Endocrine, nutritional and metabolic system	85 378	58 680 (68.7)	1.1	51 662 (60.5)	1.0
Pregnancy-related	567 492	343 822 (60.6)	3.1	271 317 (47.8)	2.5
Infectious and parasitic diseases	88 664	54 141 (61.1)	1.7	46 438 (52.4)	1.5
Mental diseases and disorders	70 739	54 678 (77.3)	0.5	47 251 (66.8)	0.4
Cancer-related	109 266	89 830 (82.2)	3.5	82 004 (75.0)	3.3
Nonemergent admissions^f^	580 715	449 191 (77.4)	14.8	398 794 (68.7)	13.5

^a^ Calculated as the total number of hospitalizations wherein the patient bypassed 1 or more nearby hospitals, divided by the total number of hospitalizations (column 1).

^b^ Includes payments to receiving hospitals for treatment of patients who bypassed 1 or more nearby hospitals. Payments are reported in 2021 US dollars.

^c^ Defined using Rural-Urban Commuting Area codes in the patient’s zip code of residence: codes 1 to 3 indicate metropolitan areas; 4 to 6, micropolitan areas; 7 to 9, small towns; and 10, noncore areas. Metropolitan zip codes were only included in the sample if they were located in a nonmetropolitan county.

^d^ Nonmetropolitan counties only.

^e^ Includes the 10 most common Major Diagnostic Category codes in the sample (first 10 rows) and a separate code for cancer-related hospitalizations. Cancer-related hospitalizations are not included in any preceding category.

^f^ Defined as nonmaternity admissions that did not originate in the emergency department and had an admission priority of urgent or elective.

**Figure.**
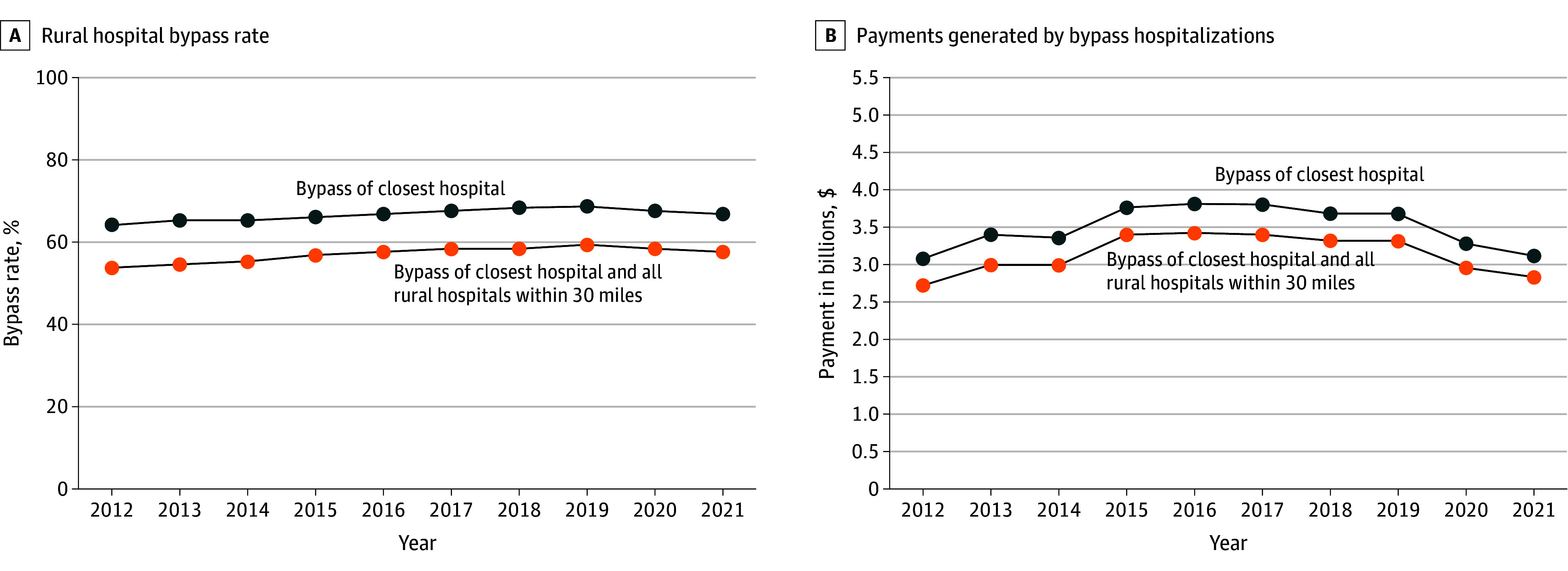
Rates of Rural Hospital Bypass by Commercially Insured Patients and Payments Generated by Bypass Hospitalizations Bypass rates are calculated as the total number of hospitalizations wherein the patient bypassed 1 or more nearby hospitals, divided by the total number of hospitalizations. Payments are reported in 2021 US dollars. Data included hospitalizations from January 1, 2012, to December 31, 2021.

There were 1 231 015 hospitalizations (56.8%) wherein patients bypassed their nearest hospital and all rural hospitals within 30 miles, accounting for $31.4 billion in payments. Variation in this broader bypass measure across rurality and clinical condition was similar to the aforementioned patterns.

## Discussion

Rural hospital bypass rates among commercially insured patients were substantial between 2012 and 2021, generating large payments to receiving hospitals. Relative to Medicare bypass rates, commercial bypass rates were high in our sample.^5^ Thus, our findings support concerns that commercial bypass contributes to financial distress at rural hospitals.

The reasons for rural hospital bypass are heterogeneous, reflecting patient choice, clinician-driven referrals, network restrictions, or limited service availability. Regardless of why bypass occurs, policymakers concerned with access to care should consider how to support critical services such as emergency care at rural hospitals where bypass contributes to low overall demand. Travel support for patients who need specialized care should also be a policy consideration.

The limitations of this study include our reliance on Health Care Cost Institute data and limited information on reasons for bypass. Also, we did not simulate payments to bypassed hospitals as if they had provided treatment; payments to bypassed hospitals will be lower than payments to receiving hospitals if bypassed hospitals have weaker leverage to negotiate prices. Further research on prices and quality at bypassed vs receiving hospitals can inform the importance of service availability at rural hospitals vs referral networks that connect rural residents to urban hospitals.

## Notes

Supplement 1. **eAppendix.** Study Population and Variable Definitions

Supplement 2.
Data Sharing Statement
