# Supplementary material for: Rural Hospital Bypass by Patients With Commercial Health Insurance
Source: JAMA Netw Open. 2026 Jan 22;9(1):e2555017. doi: 10.1001/jamanetworkopen.2025.55017 (PMC12828622; doi:10.1001/jamanetworkopen.2025.55017)
Supplement: Supplement 1. — eAppendix. Study Population and Variable Definitions [file jamanetwopen-e2555017-s001.pdf]

Supplementary Online Content

Chang JY, Carroll CE. Rural hospital bypass by patients with commercial health insurance. *JAMA Netw Open.* 2026;9(1):e2555017. doi:10.1001/jamanetworkopen.2025.55017

eAppendix. Study Population and Variable Definitions

This supplementary material has been provided by the authors to give readers additional information about their work.

© 2026 Chang JY and Carroll CE. *JAMA Network Open*.

eAppendix. Study Population and Variable Definitions

Study Population

The primary data source for this study was commercial health care claims between 2012 and 2021 from the Health Care Cost Institute (HCCI). The HCCI data is one of the most comprehensive sources of commercial data available, covering roughly 30% of Americans with employer-sponsored insurance (ESI) – approximately 40 million enrollees per year. The database contains claims from three national carriers, including over 1 billion medical claims per year from both self-insured and fully insured employer health plans. While HCCI is a convenience sample of commercial claims, it contains data on ESI enrollees across all 50 states and the District of Columbia. Each claim record includes encrypted hospital identification numbers, patient zip codes and the allowed amounts for the service (i.e., payment for care). The data also include detailed information about the type of care that was delivered, diagnoses and procedures associated with each claim, and underlying patient demographics (age group in decades, sex).

Our initial sample included hospitalizations of nonelderly, rural residents, as identified by the type of bill code in the HCCI claims (N= 3,182,071). From that sample, we excluded newborn hospitalizations (N=356,177), which are sometimes billed separately from maternal hospitalizations (though not always). We also excluded hospitalizations resulting from inter-facility transfer (N=112,620). Hospitalizations due to readmission were not excluded from our sample. We also excluded hospitalizations if the patient's zip code information was suppressed by HCCI (N = 359,110) or if we were otherwise unable to ascertain the bypass status of a

© 2026 Chang JY and Carroll CE. *JAMA Network Open*.

hospitalization (N=27,591). Of note, HCCI suppresses patient zip code when the population of the zip code is under 1,350 according to the U.S. Census, so bypass rates in this population may differ from bypass rates in our analytic sample. Finally, we excluded hospitalizations where the taxonomy of the provider did not indicate a short term, acute care hospital or hospital unit (N = 160,519). After exclusions, our sample included 2,166,054 hospitalizations. See sample selection flowchart below for a summary of our sample selection process.

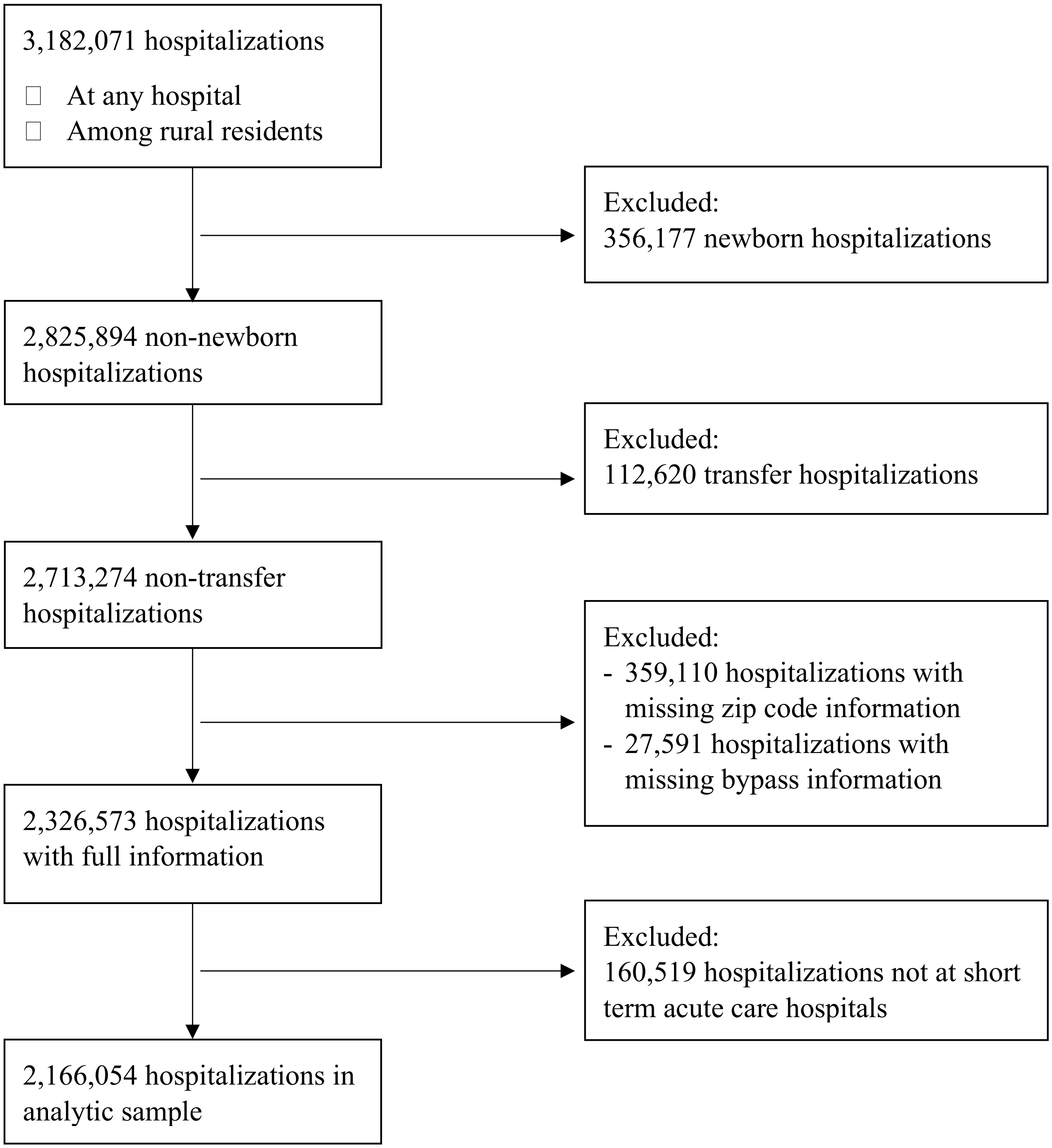

© 2026 Chang JY and Carroll CE. *JAMA Network Open*.

Variable Definitions

Rural: We defined rural areas to include nonmetropolitan counties and zip codes with nonmetropolitan Rural Urban Commuting Area (RUCA) codes, following previous work.[1] Data on county-level metropolitan status comes from the U.S. Census. Data on RUCA codes comes from the U.S. Department of Agriculture. We further used RUCA codes to define four categories of zip code rurality: noncore (10), small town (7-9), micropolitan (4-6), metropolitan (1-3). RUCA codes indicate whether a zip code is either part of an urban core or connected to an urban core through commuting patterns. Noncore zip codes (10), for example, have populations under 2,500 and have a primary commuting flow that is outside of any urbanized area. Following from our definition of rural, metropolitan zip codes (RUCA 1-3) were only included in our sample if they were located inside of a nonmetropolitan county; these zip codes account for 5% of admissions in our sample. Most admissions in our sample (74%) were among residents who lived in both rural counties and rural zip codes; the remaining 21% of admissions were among residents who lived in rural zip codes in metropolitan counties.

Bypass: We examined two measures of bypass. First, we assessed the share of patients who received inpatient care at any hospital other than their nearest hospital. Distance was measured as driving miles between the patient's zip code centroid and the hospital's zip code centroid. In the HCCI data, hospitals sometimes report multiple zip codes – for example, a claim from a system-affiliated hospital might include a zip code for the location of the treating hospital and another

[1] Carroll C, Euhus R, Beaulieu N, Chernew ME. Hospital survival in rural markets: closures, mergers, and profitability. Health Aff (Millwood). 2023;42(4):498–507

© 2026 Chang JY and Carroll CE. *JAMA Network Open*.

zip code for the location of the system headquarters. In these cases, we compute driving miles as the distance between the patient's zip code and the nearest zip code for each hospital. To the extent that the nearest zip code is incorrect, it will lead us to understate bypass rates.

Next, we examined the share of patients who bypassed their nearest hospital plus all other rural hospitals within 30 miles. This second measure of bypass allowed us to assess a broader version of bypass, examining the share of rural patients who left their market entirely for inpatient care. A notable limitation of using a mileage threshold is that it does not accommodate factors such as road ruggedness, which can make a 30-mile travel distance more or less difficult across communities. Our second bypass measure follows the definition of bypass in CMS (2020), for comparability with recent literature.[2] Note that the definition of rural in CMS (2020) differs from the definition in this paper, so comparability of estimates is imperfect.

Of note, the HCCI data do not include sufficient information to discern if hospital bypass is driven by a lack of service availability at nearby hospitals. In particular, because the HCCI do not include the census of hospital admissions, a lack of observed admissions in a particular service line at a hospital does not necessarily imply that the hospital does not provide the service (because we do not observe admissions from other payors that may fall into this service category). Likewise, we do not observe sufficient information to infer bypass driven by network restrictions or provider referrals. Thus, we present estimates of overall bypass, which combines both "avoidable bypass" (when patients bypass available services) and unavoidable bypass.

[2] Centers for Medicare & Medicaid Services. Understanding Rural Hospital Bypass Among Medicare Fee-for-Service (FFS) Beneficiaries in 2018. 2020. Accessed March 27, 2025. https://www.cms.gov/files/document/hospitalbypassamongmedicaredatahighlightsept2020-1-1.pdf

© 2026 Chang JY and Carroll CE. *JAMA Network Open*.

Previous work from CMS suggests that about a third of overall bypass can be explained by lack of service availability.[2]

<u>Clinical categories:</u> We considered clinical presentation along two dimensions. First we grouped hospitalizations into clinical categories according to their Major Diagnostic Category (MDC). Cancer-related hospitalizations were included in a separate category, with identification of cancer-related hospitalizations following Roemer (2021).[3] Second, we measured bypass among non-emergent admissions. We defined non-emergent admissions as non-maternity admissions that were not admitted through the emergency department and had an admission priority of urgent ("patient needed immediate attention") or elective ("patient's condition allowed for time to schedule"). Note that admission priority was missing for 8% of admissions.

[3] Roemer, Marc. Cancer-Related Hospitalizations for Adults, 2017. Agency for Healthcare Research and Quality. 2021. Accessed March 27, 2025. https://hcup-us.ahrq.gov/reports/statbriefs/sb270-Cancer-Hospitalizations-Adults-2017.jsp

© 2026 Chang JY and Carroll CE. *JAMA Network Open*.
