# Supplementary material for: Rural Hospital Bypass by Patients With Commercial Health Insurance
Source: JAMA Netw Open. 2026 Jan 22;9(1):e2555017. doi: 10.1001/jamanetworkopen.2025.55017 (PMC12828622; doi:10.1001/jamanetworkopen.2025.55017)
Supplement: Supplement 2. — Data Sharing Statement [file jamanetwopen-e2555017-s002.pdf]

Data Sharing Statement

Chang. Rural Hospital Bypass by Patients With Commercial Health Insurance. *JAMA Netw Open*. Published January 22, 2026. doi:10.1001/jamanetworkopen.2025.55017

Data
Data available: No

Additional Information
Explanation for why data not available: Data access is governed by a DUA with HCCI.
